# Motivations for use, identity and the vaper subculture: a qualitative study of the experiences of Western Australian vapers

**DOI:** 10.1186/s12889-020-09651-z

**Published:** 2020-10-15

**Authors:** Kahlia McCausland, Jonine Jancey, Tama Leaver, Katharina Wolf, Becky Freeman, Bruce Maycock

**Affiliations:** 1 Collaboration for Evidence, Research and Impact in Public Health, School of Public Health, Curtin University, Bentley, Western Australia 6102 Australia; 2 School of Media, Creative Arts and Social Inquiry, Curtin University, Bentley, Western Australia Australia; 3 School of Marketing, Curtin University, Bentley, Western Australia Australia; 4 School of Public Health, University of Sydney, Sydney, New South Wales Australia; 5 Present affiliation: College of Medicine and Health, University of Exeter, Devon, South West England UK

**Keywords:** E-cigarettes, Vapers, Qualitative, Identity, Subculture, Australia

## Abstract

**Background:**

Vaping is a relatively new practice, and therefore its symbolic meanings and social practices are yet to be fully understood, especially within Australia where the practice is strictly regulated. This study aimed to examine vapers motivations for use, reinforcing influences, and association with the vaper subculture.

**Methods:**

Working from a constructivist epistemology and a symbolic interaction framework, in-depth interviews were conducted with a purposive sample of 37 current (89%) and former (11%) adult vapers, 70% male, mean age of 32.5. Data was analysed via thematic analysis.

**Results:**

Vapers largely started vaping to quit smoking and underwent common experiences during their initiation phase. Subsequently, vapers tended to adopt one of two dominant identities, that of the ‘cloud chaser’ or the ‘substitute’, which some users moved between during different stages of their vaping career. The social and symbolic meaning of e-cigarettes and vaping varied and involved concepts of harm reduction, addiction, pleasure, stigma and community, and for some, connection to the vaper subculture.

**Conclusions:**

Understanding the complexities of vaping, and the nuanced differences of ‘cloud chasers’ and ‘substitute’ vapers may have important implications for health communication, research and policy. E-cigarette users within this sample were not a homogeneous group and differed in their motivations for use, association with the vaper subculture and relationship with the vape community. These findings provide new insights into the socialisation process and subsequent identity adoption of vapers within the unique regulatory environment of Western Australia.

## Background

Since entering the American market in 2007 [1], e-cigarettes have undergone a rapid evolution, with three broad classifications of vaping devices now recognised i) disposable (cig-a-like), ii) closed reusable (vape pen, pod-based), and iii) open reusable (mod) [2]. Cig-a-likes closely resemble a cigarette with a glowing tip that lights up upon inhalation and is disposed of once the e-liquid is consumed. Closed reusable systems use replaceable pre-filled cartridges which tend to be limited in functionality (i.e. inability to adjust the temperature) and were originally designed to resemble cigarettes. However, the most recent generation of closed reusable vaping devices, pod-based systems, have diverged from cigarettes and now resemble USB sticks [2]. Finally, open reusable systems comprise a refillable liquid reservoir or ‘tank’, which users fill with their preferred choice of e-liquid.

E-cigarettes were originally developed as an alternative form of nicotine delivery and potential smoking cessation device [3]. However, over the short period since their inception, they have transformed into high-tech nicotine delivery devices appealing to both non-smokers and youth [4], an outcome largely stemming from increased investment by the tobacco industry [5]. This investment has contributed to their use moving beyond their touted role as a nicotine replacement and tobacco cessation device, to a social, recreational and sensory delivery device [6] associated with new rituals and social practices [7].

Smokers cite numerous reasons for starting vaping, these include: to ease nicotine cravings and withdrawal symptoms; to quit smoking or avoid relapse; to use e-cigarettes where smoking is prohibited; reduce cost; and the belief that e-cigarettes are less harmful than tobacco [8–11]. However, recently, research has investigated the rise in ‘alternative’ e-cigarette use behaviours such as dripping (i.e. applying e-liquid directly on the atomiser) [12] and vape tricks (i.e. creating shapes from exhaled aerosol) [12, 13] which may contribute to the perception that e-cigarettes are ‘cool’ or to be used for recreation [13].

Research from Europe has explored e-cigarette user’s motivations, self-identity as vapers and involvement in vaping subcultures. Farrimond [14] identified differing motivations for use of, and varying political engagement in, vaping regimes among a sample of vapers in the United Kingdom (UK) and constructed three main typologies to describe these users: vaping for pleasure, vaping as medical treatment and ambivalent e-cigarette use, suggesting that the motives of vaping may be linked to different social identities. Similarly, a study of Norwegian vapers identified two dominant vaper identities, who Tokle and Pedersen [15] labelled ‘cloud chasers’ and ‘substitutes’. Cloud chasers were dedicated vapers who identified with symbols and values in the subculture, many of whom were politically engaged in improving e-cigarette regulation, describing a sense of belonging to the vape community. Whereas substitute vapers were former daily smokers who used e-cigarettes for smoking cessation, to improve their health, escape the stigma of smoking and manage nicotine addiction. These studies echo other international research pointing to the symbolic and identity aspects of vaping [16–19].

Vaping is a relatively new practice, and therefore its symbolic meanings and social practices are yet to be fully understood. However, it appears that through the uptake of vaping, personal and collective identities have been established and a vaping subculture has emerged [14, 15, 20]. Considering the limited extant research investigating e-cigarette use within Australia, this study aimed to examine vapers motivations for use, reinforcing influences, and association with the vaper subculture within Western Australia.

In Australia, liquid nicotine is classified as a ‘Schedule 7-Dangerous Poison’ under the Federal Poisons Standard [21]. Hence, the only legal avenue for obtaining it is through a personal importation scheme [21], which states the user must have a prescription from a physician. E-cigarettes that do not contain nicotine can be sold in some Australian jurisdictions, provided manufacturers do not make therapeutic claims. However, in Western Australia, the context of this study, it is currently an offence under the Tobacco Products Control Act 2006 [22] to sell products that resemble tobacco products, regardless of whether they contain nicotine or not.

Since the early 1990s, Governments in Australia have enacted progressive comprehensive legislation to reduce the impact of tobacco [23], and as a result, smoking rates have steadily declined. The 2019 National Drug Strategy Household Survey (NDSHS) [24] reports daily tobacco smoking rates in Australia have more than halved (11.0%) since 1991 (24.3%), and the daily use of tobacco products is most common among people aged 40–59 years (31.7%). Conversely, e-cigarette use has increased and current use is most common among those aged 18–29 (32.4%). During the time this study was undertaken the number of vape retail stores within the Greater Capital City Statistical Area (GCCSA) of Perth, Western Australia, had multiplied exponentially [25], which has resulted in increased exposure and access to these products, and perhaps reflects an increase in demand.

Vaping devices are referred to by users and scholars by a multitude of terms, including e-cigarette, ENDS (electronic nicotine delivery device), vape and mod. In this paper, the term e-cigarette is used to represent all of the various consumer products available.

## Methods

### Theoretical framework

Symbolic interactionism is a micro-level sociological theory providing the theoretical framework underpinning this study [26]. Symbolic interactionism is situated in a constructivist epistemology, focussing on the interactions between individuals rather than large scale social structures, examining how people navigate their interactions with others and allocate meanings based on their interpretation of those interactions [26, 27]. Symbolic interactionism has a history of being used to investigate drug use, the creation of deviance, and the exploration of meaning associated with new phenomena [28–30]. The symbolic interaction framework, therefore, assists in understanding a society (e-cigarette users) which is created through the repeated interactions between vapers [26, 27].

### Sampling

Participants were purposively sampled for maximum variation in demographic characteristics (i.e. sex, age, Index of Relative Socio-Economic Advantage and Disadvantage (IRSAD) - a ranking derived from the economic and social conditions of people and households within an area [31]) within the sampling frame. Data collection and analysis occurred simultaneously (March – November 2018), facilitating appropriate and targeted recruitment. Eligible participants were current and former (vaped within the last 12 months) vapers, aged over 18 years residing within the GCCSA of Perth, Western Australia [32]. Eligibility criteria were stipulated on all recruitment material.

### Recruitment

A multipronged approach to recruit participants was utilised. Recruitment flyers and posts were placed on four online vaping forums (AussieVapers, Vaping in Australia, Vaper Café Australia and E-Cigarette Forum); seven subreddits on Reddit; and 30 closed vaping Facebook groups. The lead author created personal accounts on each of the forums and social media. Facebook groups were accessed by requesting permission to enter the group as a researcher to recruit study participants. Vape retail stores, online and bricks and mortar, within the GCCSA of Perth were contacted via email, social media and webpage submission forms. Snowball sampling was also utilised.

Interested individuals were invited to contact the lead author via email or telephone to express their interest in participating and receive further details about the project and what their participation entailed. After reading the participant information statement and providing informed consent, interviews were arranged at convenient safe public locations (e.g. local café, university campus). Interviews were conducted in English by the lead author who has experience in qualitative data collection. Interviews lasted on average 49 min (range 25–86 min) and were audio-recorded with participant consent. Participants were provided with an AUD$25 gift voucher at completion of the interview as an honorarium for their time.

### Data collection

A semi-structured interview guide was developed to allow flexibly and adaptability within each interview [33], and pilot tested with two participants. The interview guide addressed the following topic areas: reasons for vaping; pathway to using; knowledge, attitudes, and beliefs associated with e-cigarette use; devices and products used; means of accessing products; attitudes of friends, family and society towards vaping and their use; and emergent subculture (see Additional file 1). As new ideas and concepts were identified within the data these data-driven concepts were fed back into the data collection process and further guided sampling and adaption of the interview guide [34]. Sampling was terminated when thematic saturation was reached [35], which was determined through the constant comparison of data with preceding data until few new themes were generated.

### Analysis and interpretation

All interviews except one (file corrupt) were transcribed verbatim by an independent professional transcription service and checked for accuracy by the lead author. The detailed notes taken by the lead author during and after interviews were sent via email to the participant the following day to review for accuracy and validation. Amendments from the participant were returned via email. Transcripts and interpretations were not provided to participants for respondent validation. Interview transcripts and detailed notes were anonymised and imported into NVivo (v12) to facilitate data organisation and linkage. The lead author conducted all coding, allowing for a single researcher to be immersed in both the data collection and analysis, thereby ensuring that the coding frame adequately described the intentions and content of the interviews [36].

The analytical process followed the steps proposed by Braun and Clarke [37] for thematic analysis and drew upon the initial and axial coding process of grounded theory [38]. The lead author played an active role in the analysis by searching for and identifying themes “to theorize the sociocultural contexts, and structural conditions, that enable the individual accounts that are provided” [37] (p. 85). Line-by-line analysis was undertaken to look for patterns of meaning and issues of interest important to the research objective and to generate a range of initial codes [38]. Codes were developed based on theoretical interest and emergent concepts that arose during interaction with and interpretation of the data. Axial coding examined the initial codes at a conceptual level to combine and connect codes to form overarching ‘candidate’ themes and subthemes in a meaningful way for the phenomenon under investigation [38]. Revision of the candidate themes then occurred at two levels. Level one involved reviewing all the data collated under each candidate theme to consider whether the data formed an intelligible pattern [37]. Some themes and sub-themes were refined during this process to create new themes/subthemes and to separate and combine others [37]. Level two involved a similar process, whereby the data were reviewed and further refined. This process, however, concerned the validity of the individual themes in relation to the data set ensuring participants meanings and voices were accurately reflected [37]. A detailed analysis was then written for each theme to report the content and meaning of patterns [37]. Working theme titles were reviewed to ensure they accurately reflected the respective analysis and the most vivid quotes were selected that best illustrated the essence of the point being described [37].

Strategies to enhance the rigour of the research included the use of a codebook to provide structure and agreement about code definitions, constructs, and themes; in-depth rich description of the research methods through field notes, personal reflections and analytic memos [39]; and discussions with team members about meaning and interpretation of findings and conceptual maps [40].

Demographic and behavioural data were analysed using descriptive statistics (SPSS v26). The reporting of this study is guided by the consolidated criteria for reporting qualitative research (COREQ) checklist [41] (see Additional file 2).

## Results

### Participants

Thirty-seven interviews were conducted with current (*n* = 33, 89%) and former vapers (*n* = 4, 11%) (Table 1), with a mean of age of 32.5 (*SD* = 7.411, range 20–45 years). Thirty-two participants (87%) were current or former cigarette smokers and five (13%) were vaping despite having never been a regular smoker. Five participants were dual users of tobacco and e-cigarettes. Former (*n* = 24, 65%) and current (*n* = 8, 22%) smokers had been using tobacco on average for 14 years (*SD* = 8.268, range 3–38 years). In comparison, participants had been vaping on average for 2.4 years (*SD* = 2.011, range 1 month – 7 years).

**Table 1:** Summary of participant’s demographic, smoking and vaping characteristics

Characteristics	Total (***n*** = 37)	Cloud chasers (***n*** = 19)	Substitutes (***n*** = 18)
**Age (years)**			
20–29	15 (40%)	8 (42%)	7 (39%)
30–39	15 (40%)	7 (37%)	8 (44%)
40–49	7 (20%)	4 (21%)	3 (17%)
**Sex**			
Male	26 (70%)	15 (79%)	11 (61%)
Female	11 (30%)	4 (21%)	7 (39%)
**Education**			
< High school certificate	6 (16%)	6 (31%)	–
High school certificate	8 (22%)	4 (21%)	4 (22%)
Technical (TAFE) certificate	14 (38%)	6 (31%)	8 (45%)
University degree	9 (24%)	3 (16%)	6 (33%)
**Employment status**			
Employed	33 (90%)	18 (95%)	15 (83%)
Unemployed	2 (5%)	–	2 (11%)
Full-time student	2 (5%)	1 (5%)	1 (6%)
**IRSAD ranking**			
Most disadvantaged	9 (24%)	5 (26%)	4 (22%)
Disadvantaged	5 (14%)	3 (16%)	2 (11%)
Median	13 (35%)	5 (26%)	8 (44%)
Advantaged	2 (5%)	2 (11%)	–
Most advantaged	8 (22%)	4 (21%)	4 (22%)
**Vaping status**			
Current vaper	33 (89%)	18 (95%)	15 (83%)
Former vaper	4 (11%)	1 (5%)	3 (17%)
**Nicotine vaping**			
Yes	26 (70%)	11 (58%)	15 (83%)
No	11^a^ (30%)	8 (42%)	3 (17%)
**Average vaping duration**	2.4 years range 0.08–7 *SD* = 2.011	2.7 years range 0.08–7 *SD* = 2.052	2.1 years range 0.25–7 *SD* = 1.971
**Smoking status**			
Current smoker	8 (22%)	4 (21%)	4 (22%)
Former smoker	24 (65%)	13 (68%)	11 (61%)
Never smoker	5 (13%)	2 (1133%)	3 (17%)
**Average smoking duration**	14.0 years range 3–38 *SD* = 8.268	13.5 years range 4–25 *SD* = 6.718	14.5 years Range 3–38 *SD* = 9.963
**Dual user**^b^			
Yes	5 (14%)	4 (21%)	1 (6%)

^a^Four participants started with nicotine, nicotine-free at the time of the interview

^b^Using both cigarettes and e-cigarettes

Two identities and approaches to vaping emerged from the data, which we labelled ‘substitute’ vapers and ‘cloud chasers’ after the dominant vaper identities constructed by Tokle and Pedersen [15]. Cloud chasing is the act of expelling large amounts of vapour using an e-cigarette, we use the term in a broader, more symbolic sense. As such, the ‘cloud chaser’ identity is formed by the experiences articulated by 19 dedicated vapers who connect with at least some aspect of the vaper subculture, whether that be engaging with hobbyist activities, the trick culture or technological aspects of vaping. Whereas the experiences of the ‘substitute’ vaper are derived from 18 vapers who primarily viewed vaping as a means to manage their nicotine addiction and quit smoking. The identity prescribed to each user was not ‘fixed’ as some participants described their movement between the two identities over time as the meaning they attributed to vaping changed (i.e. hobby to primarily smoking cessation).

### Findings

The narrative summary describes the commonalities experienced by vapers and then the experiences unique to the ‘substitute’ and ‘cloud chaser’ identity. Quotes from participants are provided in italics, followed by their pseudonym, attributed identity and age.

#### The common experience

##### “Can I have a go of that?”: an introduction to vaping

Participants were predominantly introduced to e-cigarettes through work colleagues, friends, and during their time abroad in locations where e-cigarettes were more readily available (e.g. the UK). For the majority of participants, this introduction marked the first occasion they had seen or heard about e-cigarettes. Subsequently, participants asked if they could *“have a go on that”* or the e-cigarette user suggested they try their vape. No one spoke of being pressured into trying their first e-cigarette with experimentation occurring spontaneously, either alone with the user or in the company of friends. Rarely did participants report communal experimentation where the vape was passed around to multiple people, rather it was most commonly a discrete encounter. Participants were mostly curious to try this device which in most instances functioned like a cigarette, however, were told it was not. Participants regularly reported coughing upon trying their first vape which could be attributed to several factors including the type of device (e.g. first/second versus third/fourth-generation device), device functionality (e.g. variable temperature, wattage, airflow or resistance), nicotine concentration, and the users smoking history, if any. Those who were smokers described this experience as being similar to that of their first drag of a cigarette, whereas non-smokers had difficulty articulating their experience as they had nothing to compare it to.

*“As a non-smoker, it was really hard for me to grasp the concept. Everyone was trying to explain it to me like it's either like a cigarette or a bong. I was like, ‘I don't know what you're talking about.’ They were like, ‘mouth-to-lung, or direct-to-lung’ and I still can't even understand the concept. If I had to explain to you what I do, I don't know what I do. I press the trigger, I breathe it in, it comes out.”* Karis, cloud chaser [33]

The inhalation of vapour reportedly took some time to get used to as the vapour from the e-cigarette was *“moist”* compared to the *“dry heat”* of a cigarette. Participants went on to purchase an e-cigarette for themselves after enjoying their first experience, or after disliking the encounter chose not to pursue it until another opportunity arose, years later in some instances.

##### “I was a silly teenager”: motivations for vaping

Thirty-two users were tobacco smokers for many years and described themselves as being *“very addicted”* which had resulted in several failed quit attempts. Participants had tried multiple methods to quit smoking, including going ‘cold turkey’, hypnotherapy, and nicotine replacement and drug therapies. The increasing cost of tobacco, real and feared deterioration of their health, and encouragement from loved ones to quit smoking were other significant drivers to commence and subsequently maintain vaping. For those users who had never been committed smokers, their motivations for initiating vaping were varied and included socialisation with other vapers, to appease food cravings, and as a diversion from alcohol or illicit drugs.

##### “On their way to their vaping journey”: starting off

Most participants opted to start their *“vaping journey”* with a disposable (cig-a-like) or pen-style closed-system device which introduced them to vaping at a rudimentary level, as explained by River [42] *“… something basic, where you push the button, you suck on it…”*. Most, however, described these devices as unsatisfying (e.g. mute flavour, inadequate throat hit), poorly fabricated and not producing enough vapour. Subsequently, most participants progressed to an open-system device which provided functionalities to enhance and personalise their flavour profile, adjust temperature, voltage, resistance, airflow and nicotine concentration, and comprised a refillable tank and rechargeable batteries. These features were particularly important for tobacco smoking participants, and were conducive to replicating the *“throat hit”* they were accustomed to.

*“It [e-liquid] had no nicotine in it, as is Australian rules. I ended up actually putting my own [nicotine] in it because it was just, too smooth basically, you could taste it, but you couldn't feel it which is what I want, I want to feel it [throat hit].”* Brody, substitute [34]

Transitioning to vaping from *“analogue cigarettes”* was a daunting process for some, and more so for those who had never been committed smokers. Participants explained that other vapers had tried to describe to them how they were required to inhale to achieve the desired throat hit and experience the best flavour.

*“My friends were kind of just like, ‘Just pull it in. You'll cough if you do it hesitantly.’ I was super scared of just going-- [inhales], on this thing that's firing. The first few times that I did it, I don't think I was doing it properly because I was firing it for a really short amount of time, taking like really small puffs. Then when I did do it properly, I was like, oh this is what it's meant to feel like and taste like."* Karina, substitute [24]

For those who were smokers, they described the inherent challenges, actions and processes of transitioning from cigarettes to e-cigarettes which took perseverance, including no longer lighting a cigarette, maintaining a charged device, importing, mixing and storing liquid nicotine, and accepting the physicality of the device compared to the slender profile of a cigarette.

“Filling it up, charging it up, carrying it around, because it f***ing weighs a ton, as well. It just became too hard filling it up. It was always leaking, and it's not as simple as clicking a button and smoking it. You have to set it to what you want and all that.” Jonathan, substitute [27]

##### “A bit of a learning curve”: gaining knowledge and understanding

The majority of users reported being proactive in conducting intensive ‘self-learning’ through the internet and social networking platforms, other vapers, and to a lesser extent, retail stores, to acquire relevant skills (i.e. nicotine strength, mixing and safety; steeping; inhalation style *“direct-to-lung”* or *“mouth-to-lung”*; building and changing coils), information on health and safety, the meaning of vaping specific language and jargon, and troubleshooting techniques. Vape forums, social media (i.e. Facebook) and content sharing platforms (i.e. YouTube) were usually the first resources accessed to acquire knowledge and support. Participants reported simultaneous active (i.e., contributing content) and passive (i.e., viewing content posted) engagement in multiple local and international vaping groups and forums to discuss personal experiences, exchange information, and obtain new knowledge. Passive engagement provided newcomers with an opportunity to observe the online community and its rules, whereas more established vapers kept their *“finger on the pulse”* by monitoring the rise of new products. Conversely, active contribution to these fora provided opportunities for learning among ‘newbies’ and mentorship for more experienced vapers.

“Well, that's where I got most of my knowledge from [online vaping groups]. It's hard to explain, it's a little family sort of thing, like whether it's starting off fresh or you know plenty of s**t you can always go there, get the advice that you need. That's definitely a helpful thing as well. … You've got people in those groups that have been vaping for 10 or so years, like starting off when it was just a tiny industry, a fresh industry. Then you've got people that are trying to get off the smokes and they ask for advice from there.” Timothy, cloud chaser [20]

##### “There is something for everyone”: finding the right product

During the transition phase from cigarettes to vaping, some respondents reportedly struggled to quit smoking, relapsing on occasion, and/or dual using with cigarettes until they found the right combination of flavour, nicotine concentration and device. Finding the right combination was the moment many sensed they could quit smoking, citing the sophisticated features of the second (closed reusable) and third-generation (open reusable) devices most effective for smoking cessation.

*“For me, it was getting used to the action but also … finding the right flavours and then it was getting the nicotine level right, then it was finding the right device that was going to satisfy my intake, my draw in and my exhale… I’d buy one and go, ‘Oh it’s a bit crap. I still want a cigarette. Why do I still want a cigarette?’ Then eventually I found another device which was a bit more powerful and I found flavours that I actually liked and so when I found that flavour I can tell you it was 48 hours between finding this particular flavour blend to when I had my last cigarette.”* Ursula, cloud chaser [43]

##### “I haven’t quit I’ve upgraded”: from one addiction to another

Smoking cessation was viewed very differently to nicotine cessation, with the majority of users opting to continue using nicotine in their vapouriser to keep them from relapsing to cigarettes. Three of the five users who had never been committed smokers chose to use nicotine in their vapouriser after being introduced to it by a romantic partner or friend. All three users described themselves as not being addicted and that they could easily give it up if they tried. The end goal for many participants was not to be reliant on nicotine at all, and they explained how they were, or had, implemented strategies to reduce the concentration of nicotine they were using, such as alternating between nicotine and nicotine-free vapourisers and gradually tapering down the nicotine concentration. Four users had successfully quit smoking and were continuing to vape nicotine-free. Those users who were happy to continue to use nicotine in a *“cleaner”* and *“healthier”* form (e-cigarettes), or felt they were not ready to *“wean”* themselves off for fear of relapsing to smoking, recognised that they had completely transferred the behavioural and sensorimotor aspects of smoking to vaping. However, they believed because they were no longer smoking any potential ill-health inferred by vaping seemed inconsequential.

*“As it stands, I have no intention of stopping [vaping] because whilst I'm using that [vapoursier] I have no intention to have a cigarette. That's how passionately I don't want to smoke again, but I'm not prepared to risk it at this time, but who knows? … Do you know what, I often think I probably do need to cut back a little bit and I think, well I'm not smoking?”* Ursula, cloud chaser [43]

Previous cigarette users observed that vaping fit nicely into their routine, which was once occupied by cigarettes (i.e. driving, coffee). Vaping allowed them to continue to enjoy the social aspects of smoking (i.e. drinking alcohol) and to placate feelings of stress or anxiety.

*“Yeah, I still make a point of, especially when I first quit, of keeping that routine of going outside to smoke, or vape, just so it felt a little bit more like I was having a cigarette. It wasn't such a drastic change. You know like straight after a meal or things like that, my trigger moments. I would still get up, keep it to that little bit of a routine. Get up, go outside, have my vape, go back inside.”* Ella, substitute [41]

Socialising with other vapers and smokers was said to reinforce and maintain their use of e-cigarettes. Even amongst those who were never committed smokers and those who were now vaping nicotine-free.

##### “I’m not a smoker. I’m a vaper”: breaking free of tobacco

In general, users referred to their device as a vape, themselves as a vaper, and the practice as vaping. Some felt the term ‘e-cigarette’ too closely aligned with smoking discourse and supported associations with negative connotations of death and disease. Vape products were generally not considered to be tobacco products, especially with the evolution of vaping devices and how they no longer resembled a cigarette, as earlier generations had.

“They need to stop calling them e-cigarettes because they're not cigarettes. That s**ts me up the wall, they're not cigarettes.” Ian, cloud chaser [29]

Several users documented how they had experienced the *“ignorance”* of both smokers and non-vapers, and many seized the opportunity to *“educate”* these people. They highlighted the features which distinguished vaping from smoking, such as not containing tobacco and the production of vapour, not smoke, and the perceived positive changes to their health they had experienced since starting vaping, in the hope of reducing the stigma and the estrangement they felt.

*“… people will say, ‘why don't you just smoke cigarettes?’ which I think is a strange thing to say. People just misunderstanding the health risks.”* Julia, substitute [26]

For some, they could not escape their internalised feelings of smoking-related stigma, and as a result, avoided vaping in public.

*“I generally try not to vape in public because it is not stealthy unless you're using a little stealthy device … People can see you a mile away, and I get really embarrassed. But I used to hide when I was a smoker as well. At least when I was a smoker I could hide in my car. Even with my vape, I get in my car and there's big clouds coming out.”* Ursula, cloud chaser [43]

#### The substitute vaper

##### “A means to an end”: Vaping to quit smoking

For those ‘substitute’ vapers who were former smokers, they viewed their vaping experience as a practical means to quit smoking and valued the positive effect vaping had on their health and wallet. They were aware of the existence of more enthusiastic vapers, however, at the time did not associate with the vaper subculture as ‘cloud chasers’ did.

*“I see people, and it's kind of a sport for them, they make big clouds … I don’t really buy into that. … It's not where my mindset is. For me, yeah it [vaping] really is a means to an end [nicotine/smoking cessation].”* Ella, substitute [41]

##### “It’s just a revolving circle”: stigma

Although many acknowledged the stigma they had endured as a smoker in Western Australia, some vapers holding the ‘substitute’ identity now projected these same negative feelings to fellow vapers associated with the ‘cloud chasers’ subculture, perpetuating the circle of stigma.

“People think that people smoking vapes think they’re ‘cool’. Sitting in their car and they've got big clouds coming out of the car. Even I do it. When I see clouds like that I think, ‘You d***head. You think you’re cool vaping like that?’ … Like I’ve seen the way people blow out their clouds I’m like, ‘You’re one of these d***heads who’s overclocking the battery’ … .” Milo, substitute [36]

##### “It is not stealthy”: managing vaping in public

Vaping is notorious for producing large vapour clouds (although some products such as JUUL are very discrete), and as such some ‘substitute’ vapers spoke of how they disliked the attention vaping brought them from bystanders, and spent energy devising strategies to manage their e-cigarette use in discreet ways, such as vaping alone. This was especially pertinent for some young women:

*“It is a bit showy because like there's a lot more vapour. I guess the only place in public that I do it and feel kind of safe is like just at the park when I'm taking a walk or something.”* Karina, substitute [24]

#### The cloud chasers

##### “I’ve gone full enthusiast”: the vaper subculture

This group of respondents shared the view that e-cigarettes are a healthier alternative to smoking, however, more importantly, vaping also offered social and symbolic functions not provided by *“analogue cigarettes”*.

Vaping was differentiated from smoking, with some describing it as a hobby, which at times could be all-consuming and expensive. Nevertheless, many genuinely enjoyed customising their experience through the collection of various flavoured liquids and coloured devices, experimenting with the creation of their own juices, engaging in the technological aspects of vaping and building accessories, such as coils.

*“I play around with them [making coils], I do all my own, I build all the things, I use all the rebuildable stuff. So yeah, it has become a bit of a hobby, which is why I think it appeals to certain people, because it has that sort of community aspect where it becomes like a hobby … they all sort of get together…”* Wade, cloud chaser [28]

A minority of vapers reported attending *“build days”* and *“vape meets”* where users got together to learn about Ohm’s law and battery safety, how to build coils, and to meet new people and socialise, as the Western Australian vape community was reportedly not as established as others in the Eastern States of Australia.

Participants commented on the various ‘types’ of vapers (i.e. hobbyist, flavourist) and ‘levels’ (i.e. novice, advanced user, expert, veteran) one could progress to. Participants categorised themselves by comparing their preferences and level of experience with others, which was influenced by various factors including vaping duration, type of device they were capable of safely using (regulated vs unregulated (no circuit board and runs directly off a battery)), possessing an online profile or presence, and experience in the retail industry.

*“[I’m] close to the expert stage. An advanced user, I'd say. When you start using mechanical mods, that's when you're an advanced user.”* Zadie, cloud chaser [27]

*“I've gone full enthusiast … I want to have the experience. I'm also hoping to get a job in one of the vape shops in Perth because I'm really enthusiastic about health or being able to help people.”* Quade, cloud chaser [24]

A small proportion of ‘cloud chasers’ were highly immersed in the vaping subculture and were actively involved in, or managed specialised vape groups, provided product reviews to YouTube, Instagram and Facebook, and some were even ‘sponsored’ by local or international e-cigarette brands to promote their products on social media. Relationships between these vapers and their sponsors were established by one of them contacting the other, usually through social media.

*“I'm part of this group called Cloud Kings Australia. Cloud Kings are basically all over the world. There's a group of them in Sweden, Mexico, Germany, France, Amsterdam, mostly in Europe. We get sponsored by companies, get free product from those companies, and then we rep[resent] those companies.”* Zadie, cloud chaser [27]

Few were also deeply entrenched in the vape trick culture:

*“Absolutely, there's an absolute technique [to vape tricks]. We've got it down to a really fine art. There's names of [tor]nados that you can do like specialised ones and stuff like the DNA, the double, oh it’s crazy. So we go all out. Like you've got to wet the table, make it stick, and you've got to layer it. So we do layer upon layer upon layer of smoke. No one’s allowed to breathe. If you breathe, you're dead.”* Clara, cloud chaser [33]

##### “Vaping brings people together”: for the cause and the community

The vape community, especially the online community, was described as *“free of judgement”* and provided for many a sense of connection and belonging. Participants described how their communication with like-minded vapers gave them the forum and permission to *“nerd out”* and voice their struggles and triumphs with a group who they felt would listen and be responsive, which some users did not feel they were able to do with their non-vaping friends and family. For those who were more experienced vapers, they felt it was important to give back to the community and chose to mentor new vapers through the initiation process.

*“I'm in a lot of Australia-wide groups ... and it’s community-minded. … It's a way to quit smoking, sure, it's a health choice, but it's also a hobby for a lot of people, so I think these groups are both support networks and hobbyists. … I think it is important because there's nowhere else to get that support to quit smoking. For me that's what vaping is all about, it's about quitting smoking and staying off the cigarettes. … For people like myself who have tried everything … It is important for me to give back, so I give a lot of advice to people that say ‘Hey I don't know what to do.’ I try and give people the advice that I didn't get but also just making friends Australia-wide, getting to know people. It's awesome. It's a pretty cool community, yeah.”* Ursula, cloud chaser [43]

Further, some participants had turned vaping into a business; were currently working, or aspiring to work in the retail industry; or were creating a social media presence (i.e. reviewing products, seeking sponsorship) for themselves. Some of these participants who were heavily involved in the online community and/or retail industry expressed frustration with the *“childish”* and *“bitchy”* behaviour displayed by some of the vape community online, especially among local and inter-state retailers. Instances of online users *“dobbing”* on people to the authorities who were selling nicotine and/or devices were described, as well as general unsocial behaviour as illustrated by one local business owner:

*“They're [vape retailers] just very childish, … and because it's still quite a small community, everything's a personal attack against someone else. Like, if so and so were to have a sale and then he'd think that it was a direct attack on him. It is very clicky and very immature a lot of the time, I don't know why. I don't really bother doing much with Facebook groups because that’s just where it all is. When it's in-store and stuff and it's all very professional, everyone's very eager to help, it’s just everyone seems to become a keyboard warrior online.”* Wade, cloud chaser [28]

Users who heavily invested in the culture or hobbyist ethos were inclined to perceive their device as an accessory, or a status symbol which was dependent on having the very latest and greatest device. For these vapers, vaping not only encompassed their passion and desire to help others quit smoking but their livelihood, which now strongly aligned with their core values.

*“All my life I've had trouble [working] in retail because I have an ethical code where I can't sell something that I don't believe in and I believe 100% in the industry of vaping and what their motives are. I think it's good. It is entirely good and all the people that I've met who also promote it and stand behind it have good intentions, and their sole drive is to see people get well and stop smoking. We want to make smoking history just as much as the non-smokers. That's the thing … almost all vapers are reformed smokers…”* Quade, cloud chaser [24]

## Discussion

The Australian NDSHS has been regularly conducted since 1985, and first provided limited data about e-cigarette use in 2013. Data from the most NDSHS [24] reports the most prevalent e-cigarette users are male current and former smokers, which is reflective of our sample population. Data does not distinguish whether users use nicotine in their vapourisers, nor what type of device they use. Enhanced surveillance and reporting of e-cigarette use within Australia would contribute to a deeper understanding of the population using e-cigarettes, the reasons for using and devices used among this cohort, and would assist policymakers to determine where public health efforts should be focussed.

Thirty-two vapers in this sample were committed smokers for several years and five participants were dual users of tobacco and e-cigarettes. The primary reason for initiating vaping was to quit smoking, citing less than optimal successes with other TGA (Therapeutics Goods Administration^1^) approved smoking cessations aids, as also described by a sample of American vapers [44]. Vaping was considered more satisfying and therefore more supportive of successfully quitting smoking compared to other methods due to its similarity with conventional smoking, namely the inhale and exhale of vapour, nicotine hit, and the hand-to-mouth action, as also documented in other international research [16, 18, 43]. Furthermore, vaping does not expect one to relinquish the rituals and habits connected to smoking [45]. The conclusions surrounding the effectiveness of e-cigarettes as a smoking cessation aid and their harm reduction potential, however, are varied and depend on several factors, such as whether the smoker switches completely to e-cigarettes, becomes a dual user with cigarettes, and whether the user becomes a sustained and persistent vaper [42].

The majority of vapers in this sample were former smokers, however, several respondents had taken up the practice despite having never smoked. Understanding how vapers ‘make sense’ of their health practices [46] is required to understand the processes by which vapers make health behaviour choices, such as choosing to vape, so that appropriate tailored communication on the risks and benefits of e-cigarette use can be developed [47]. Limiting vaping uptake by non-smokers is essential and the supportive role Australia’s strict regulation plays in limiting this uptake and exposure to marketing is discernibly apparent when compared with vaping prevalence within countries with more liberal regulation (i.e. United States (US) [48, 49] and UK [50, 51]).

Participants within this study generally exhibited limited knowledge of the potential health effects of e-cigarettes. However, as reported by vapers abroad [52, 53], they expressed many positive attitudes towards e-cigarettes, held very strong opinions that vaping offered them an alternative means to consume nicotine, and based their decision to use e-cigarettes on perceived harm reduction compared to cigarettes. For them, the individual health benefits experienced and the tangible sense of satisfaction since ceasing smoking outweighed the potential health risks of maintaining vaping. Furthermore, continued nicotine addiction was largely perceived as unproblematic so long as it helped maintain a cigarette-free lifestyle, also documented by others [43, 54]. This concept has been studied by Oakes and Chapman [55] who explored the rationalisations smokers use to explain their justification of continued smoking and suggest a series of self-exempting beliefs may provide smokers with a false sense of security and ultimately block them from exploring the importance of quitting. Given the complexity of nicotine and addiction, and the assortment of information presented on e-cigarettes, it is not unexpected that users in this study and overseas [56] rely on their own experiences, and that of others, to inform their behaviour and decision-making processes [18]. This highlights the need for accessible, clear and impartial information about e-cigarette use which communicates the benefits, risks and current uncertainties to health professionals and the public about e-cigarettes [43] and continued support for nicotine cessation through approved cessation methods.

Participants mostly described positive reactions from friends and family to their e-cigarette use, particularly when their goal was to abstain from smoking. In this sample of vapers, few had close friends who vaped and therefore sought camaraderie through online fora and vape retail stores. As found in other qualitative inquires [16], the notion of a vaping community was recurrent. However, participating in a community that accepts the practice may make it difficult for individuals to quit and therefore contribute to sustained use [57]. These findings suggest that social norms surrounding e-cigarette use have a potentially powerful influence on initiation and maintenance and that understanding social networks is integral to prevention efforts.

Although tobacco smoking is legal in Australia, the decline in prevalence combined with the denormalisation of smoking and societal aversion has fated the behaviour to be predominantly relocated to the fringes of society and viewed as a deviant and marginalised behaviour [58]. For some smokers in this study, feeling stigmatised for being a tobacco smoker was the catalyst for them to redefine themselves as ‘vapers’, as supported by findings from Barbeau and Burda [16], making the language used (i.e. not referring to vaporises as e-cigarettes) incredibly important in an attempt to escape the stigma attached to cigarette smoking [44]. This redefinition and transition from smoker to non-smoker has been argued to play a key role in supporting successful smoking cessation [59]. However, through the quest to obtain the socially desirable non-smoker status, smokers have adopted another behaviour that maintains addiction and deviates from current societal norms, an unapproved and unconventional means to quit smoking.

Two approaches to vaping emerged from our data, that of the ‘cloud chaser’ and the ‘substitute’. Vapers within this sample displayed similar subcultural elements and practices to those reported in the international literature examining the motivations of e-cigarette users, identity formation and involvement in the vaping subculture [14, 15] which could be diffused via global structures such as social media. However, some subcultural elements are localised to Australian vapers due to the unique social conditions under which the behaviour has evolved. For example, the vaping subculture which has emerged in the US is more encompassing than in Australia, which may be attributed to differences in the countries regulatory contexts [60], access to nicotine products, and exposure to mass marketing [61] and subcultural practices (e.g. vaping conventions [62] and abundant vape stores [63]).

Supported by Farrimond [14] and McQueen and Tower [64], ‘cloud chasers’ perceived their affiliation and connection with the vape community in the online and offline milieu as a positive source of support and reinforcement. Moreover, vaping was regarded as an integral part of their social identity, influencing how they behaved and the social and political activities they engaged in. Given the loss of identity and social engagement reported by individuals who quit smoking, the social opportunities, and group and community experience of vaping may be a particularly appealing aspect of the endeavour [6, 16, 53]. Furthermore, vaping was explicitly differentiated from cigarette smoking and referred to by many ‘cloud chasers’ as a hobby. Several dimensions of ‘pleasure’ were identified, including the sensory experience (i.e. flavours) and electronic and technological aspects of vaping [6, 65]. Such descriptions of enjoyment are not usual in the substance-use discourse [66] due to the dominance of the ‘pathology paradigm’ which marginalises the idea of pleasure concerning drug use [67].

The assessment that e-cigarettes are a tool to manage nicotine addiction among ‘substitute’ vapers may explain why these users did not strongly identify with, or actively rejected connection with the social identity of vaping, and enjoyment did not play a substantive role in their use and maintenance [14]. Research suggests that cessation goal-oriented vapers may be less likely to become persistent e-cigarette users compared with vapers who do not stipulate future intentions to quit [68, 69]. The nuanced differences in experiences of ‘cloud chasers’ and ‘substitute’ vapers may, therefore, contribute important insights for health communication. Australia has implemented a suite of effective strategies [23] to combat tobacco smoking that could be applied to e-cigarettes, such as supplementing health communications with legislation (e.g. health warnings, plain packaging, smoke-free laws that include e-cigarette use), until there is scientific evidence regarding their safety and efficacy as a tobacco cessation therapy [70].

E-cigarettes are both technically complex devices, which novice users may find difficult to spontaneously start, and a non-medical consumer product, which has resulted in the need for many aspiring users to look to other vapers as their experts, building a vast and international social network of shared knowledge and identity [14]. A common experience among this cohort of vapers was their use of e-cigarette forums and social media groups to discuss personal experiences, exchange information, and obtain new knowledge, similarily reported by vapers in New Zealand [18]. Seasoned vapers and newcomers disclosed periods of both active and passive engagement (also known as ‘lurking’ [71]). Lurking served newcomers with an opportunity to observe the community and its rules [72], whilst it provided more established vapers with the opportunity to monitor changes in the industry and the development of new products. Conversely, active contribution to these fora provided opportunities for learning among ‘newbies’ and mentorship for more experienced vapers. Some research suggests that joining and actively participating in e-cigarette-related social media communities [13, 73, 74] may play an important role in the development of ones vaping identity [14, 18] and can exert a significant influence on attitudes and behavioural intentions toward e-cigarettes [75]. The investigation of dedicated vaping fora, therefore, may be valuable to study interactions among users and how these interactions shape e-cigarette knowledge, attitudes and behaviours.

These findings were gathered from a small purposive sample within a specific geographical context and time, and therefore may not be generalisable to the broader vaping community or e-cigarette users abroad due to Australia’s regulatory environment, absence of mass media advertising and lack of Government endorsement as a smoking cessation aid [76]. However, the consistency with other research suggests our findings are not atypical. All participants in this study were adults, therefore these results may not be generalisable to younger vapers.

## Conclusion

Few studies have explored vapers motivations for use, reinforcing influences, and association with the vaper subculture, especially within the unique regulatory context of Australia. We found that our sample of vapers largely started vaping to quit smoking and underwent common experiences during their initiation phase. Subsequently, vapers tended to adopt one of two vaper identities, that of the ‘cloud chaser’ or the ‘substitute’, which some users moved between during different stages of their vaping career. The social and symbolic meaning of e-cigarettes and vaping were diverse. ‘Cloud chasers’ connected with the vaper subculture in varying degrees and involved concepts of pleasure, community and performance. However, the aesthetic and performance part of the subculture, in particular, had little appeal to ‘substitute’ vapers who largely viewed their use of e-cigarettes as a means to quit smoking, and enjoyment did not play a substantive role in their use. Understanding the complexities of vaping, and the nuanced differences of ‘cloud chasers’ and ‘substitute’ vapers may have important implications for health communication, research and policy. Our findings add to the understanding of the varying motives for use and provide new insights into the socialisation process and subsequent identity adoption of Western Australian vapers.

## Supplementary information

**Additional file 1.** Data collection guide. The data collection guide includes the information recorded about the interview, participant’s demographic and behavioural information and the interview guide.

**Additional file 2.** COREQ checklist. A checklist of items that should be included in reports of qualitative research.

## Data Availability

The datasets used and/or analysed during the current study are available from the corresponding author on reasonable request.

## Abbreviations

E-cigarette: Electronic cigarette
ENDS: Electronic Nicotine Delivery Device
COREQ: COnsolidated criteria for REporting Qualitative research
GCCSA: Greater Capital City Statistical Area
IRSAD: Index of Relative Socio-Economic Advantage and Disadvantage
NDSHS: National Drug Strategy Household Survey
TGA: Therapeutic Goods Administration
US: United States
UK: United Kingdom

## Footnotes

1 The Therapeutic Goods Administration is the regulatory body for therapeutic goods in Australia and is responsible for conducting assessment and monitoring activities to ensure that therapeutic goods available in Australia are of an acceptable standard and that access to therapeutic advances is in a timely manner.
