# Supplementary material for: Motivations for use, identity and the vaper subculture: a qualitative study of the experiences of Western Australian vapers
Source: BMC Public Health. 2020 Oct 15;20:1552. doi: 10.1186/s12889-020-09651-z (PMC7559168; doi:10.1186/s12889-020-09651-z)
Supplement: Supplementary file 1 — Additional file 1. Data collection guide. The data collection guide includes the information recorded about the interview, participant’s demographic and behavioural information and the interview guide. [file 12889_2020_9651_MOESM1_ESM.docx]

**Interview information**

ID number:
Location of interview:
Date:
Start time:
End time:

**Participant demographic information**

Gender:
Age:
Country of birth:
Suburb:
Highest level of education completed:
Employment:
Current e-cigarette user, how long?:
Past e-cigarette user, how long?:
Current tobacco user, how long?:
Past tobacco user, how long?:

**Interview guide**

**Reasons for use**

- Why did you start using e-cigarettes?
- Do you currently smoke tobacco as well as using e-cigarettes or have you ever used both at the same time?
- Do you still get cravings for cigarettes?
- Have you had any positive effects since using e-cigarettes? (health, physical, psychological, social)
- Have these experiences contributed to your continued use of e-cigarettes?
- Have you had any negative effects since using e-cigarettes? (health, physical, psychological, social)
- Have these experiences considered you to think about giving up using e-cigarettes?
- Have you thought about stopping e-cigarette use? Or why did you stop using e-cigarettes?
- Is there anything that would potentially make you stop using e-cigarettes?
- Do you use e-cigarettes in front of people who don’t smoke traditional cigarettes or e-cigarettes?
- Has the increase in the price of traditional cigarettes influenced your decision to use e-cigarettes?
- If the price of e-cigarettes were to increase would this influence your decision to continue using e-cigarettes?

**Pathway to using e-cigarettes**

- How did you start using e-cigarettes?
- How long ago did you first start using e-cigarettes?
- Can you explain the scenario the first time you used an e-cigarette?
- What made you want to try an e-cigarette?
- How did you learn to use e-cigarettes?

*If someone taught them*

- Did you approach this person to teach you?
- Did someone else ask on your behalf?
- Did someone approach you?

*If they chose someone*

- Why did you choose this person to teach you?

*If someone asked on their behalf*

- Who was the person that asked on your behalf?
- Why did the person who asked on your behalf ask this certain person to teach you?

*If someone approached them*

- In what setting did this occur?
- Did you feel pressured to say yes?
- What relationship did you have with the person who taught you?
- Did you look up to the person who taught you?
- What were the techniques of use that you were taught?

*Through observation*

- Who were the people you observed?
- What were the techniques of use that you observed?

*Self-taught*

- Can you explain the process you used to teach yourself?
- Were any online tutorials used?
- Have you ever taught someone else to use an e-cigarette?
- Can you explain the process you used to teach someone else?
- Do you tend to use e-cigarettes when you are alone, or is it more of a social activity with others?
- Are there times when you are more inclined to use an e-cigarette? E.g. when you are stressed?
- Does using an e-cigarette help you when you feel like this?
- When you use e-cigarettes how do you feel? E.g. mood, physical effects
- How often do you spend time with people who use e-cigarettes?
- Who are the people that you spend time with that use e-cigarettes? E.g. parents, brothers or sisters, friends, sports teammates
- How would you describe your relationship with these people? E.g. friendly, fleeting, permanent, competitive

**Knowledge, attitudes, and beliefs associated with e-cigarette use**

- Can you tell me how you first became aware of e-cigarettes?
- Do you believe that e-cigarettes are better for your health than traditional cigarettes?
- Do you think the vapour from e-cigarettes can be classified as second-hand smoke?
- What are your thoughts on using e-cigarettes to quit smoking?
- Do you think e-cigarettes should be registered as a quit smoking aid?
- What do you think about people using e-cigarettes if they have never smoked traditional cigarettes before?
- Do you think e-cigarettes have the potential to normalise tobacco smoking again?
- Do you agree with the notion that e-cigarettes might be a gateway to tobacco cigarettes for non-smokers?
- Do you think there is the same sort of stigma attached to e-cigarettes as there is to traditional cigarettes?

**Devices and products used**

- What type of e-cigarettes do you use?
- What type of e-cigarette did you first start using?
- What type of e-cigarette do you use now?
- How did you learn to use this type of e-cigarette?
- Have you tried different flavoured e-liquids?
- What flavour is your favourite?
- Have you ever made e-liquid?
- Have you ever experienced different effects when trying different flavours?
- Have you ever had an adverse reaction to a certain e-liquid or flavour? E.g. Throat irritation or nausea?

**Means of accessing product**

- How and where do you buy e-cigarettes and e-liquid?
- Do you tend to purchase your e-cigarettes and e-liquid online or at a brick and mortar shop?
- Are you able to tell me some of the websites you use or shops you attend?
- How did you first come across this site/shop?
- Do specials and e-cigarette promotions encourage you to buy more?
- How do you find out about other sites/shops to browse?
- Do you use e-liquid that contains nicotine?
- From your experience how easy or difficult has it been to purchase e-cigarettes and e-liquid online/from a shop?

**Attitudes of friends, family and society towards vaping and their use**

- Do your parents and/or friends know that you use e-cigarettes?
- If no, have you deliberately kept it from them?
- If yes, how do they feel about your use?
- Do you think your parent and/or friends attitudes or opinions towards smoking tobacco/using e-cigarettes have influenced your decision to use e-cigarettes?
- Have you ever used an e-cigarette in a public place?
- Have you ever been asked to stop vaping in a public place? How did this make you feel?
- Do you think people have the right to ask you to stop using e-cigarettes in public places?
- How do you feel about having to vape in ‘smoker’s areas’? Especially for those trying to quit, and for having to inhale second-hand smoke.

**Emergent subculture**

- Are you currently, or have you ever been part of any e-cigarette groups or communities? This can include online groups
- How did you initially get involved in this group?
- What does it mean to be a part of this group?
- Do you know of any vaping or e-cigarette gatherings or events in Australia?
- Have you ever attended one of these events in Australia? What about overseas?
- How did you find out about these events?
- Do you like to customise your e-cigarettes?
- Can you tell me more about your process of customisation?
- Why do you customise your e-cigarettes?
- How did you learn to customise your e-cigarettes?
- Do you watch vape videos online or on social media? E.g. cloud trick, cloud chasing, product reviews
